# Purebred dogs show higher levels of genomic damage compared to mixed breed dogs

**DOI:** 10.1007/s00335-023-10020-5

**Published:** 2023-10-21

**Authors:** Alfredo Santovito, Martina Saracco, Manuel Scarfo’, Alessandro Nota, Sandro Bertolino

**Affiliations:** Department of Life Sciences and Systems Biology, University of Turin, Via Accademia Albertina 13, 10123 Turin, Italy

## Abstract

Inbreeding is a common phenomenon in small, fragmented or isolated populations, typical conditions of many threatened species. In the present paper, we used a new non-invasive approach based on the buccal micronucleus assay to evaluate the possible relationships between inbreeding and genomic damage using the dog as model species. In particular, we assessed the frequencies of micronuclei and other nuclear aberrations in a group of purebred dogs (*n* = 77), comparing the obtained data with those from a control group represented by mixed breed dogs (*n* = 75). We found a significant increase of micronuclei, nuclear buds and total nuclear aberrations frequencies in purebred dogs compared to mixed-bred dogs. The absence of significant differences in the frequency of micronuclei and other nuclear aberrations amongst different breeds reinforces the hypothesis that the observed increased genomic damage amongst purebred dogs may not be due to a different genomic instability typical of a particular breed, but to inbreeding itself. This hypothesis is further confirmed by the fact that other endogen confounding factors, such as sex, age and weight, do not contribute significantly to the increase of genomic damage observed amongst purebred dogs. In conclusion, results presented in this study showed that, in purebred dogs, inbreeding may increase the levels of genomic damage. Considering that genomic damage is associated with increased physiological problems affecting animal health, the results we obtained may represent a stimulus to discourage the use of intensive inbreeding practices in captive populations and to reduce the fragmentation of wild populations.

**Supplementary Information:**

The online version contains supplementary material available at 10.1007/s00335-023-10020-5.

## Introduction

Inbreeding is a common phenomenon in small, fragmented or isolated populations, and it is typical of many threatened populations (Björklund 2003; Wright et al. 2008). Inbreeding could reduce individual fitness, reproductive success and lifespan and increase susceptibility to environmental stress (Chu et al. 2019). A high level of inbreeding in a small population is associated with a loss of genetic diversity, inbreeding depression and the spread of deleterious alleles (Lewis et al. 2015). As a result of the reduced fitness and ability to respond to a changing environment, populations are at risk of extinction. Several studies have analysed the impact of inbreeding depression using different genomic approaches, including genome-wide association studies (Kropatsch et al. 2015; Melis et al. 2013). Another possible effect of inbreeding could be an increase in the frequency of genomic damage. This damage occurs during cell division, such as mutations, translocations, or telomere shortening and is not repaired (Thomas et al. 2009; Bolognesi et al. 2013).

Although it is crucial to understand how inbreeding can affect fitness in small and inbred populations, it is challenging to assess such a consequence in wild populations, mainly due to the logistic problems of data collection (Björklund 2003). Evaluating the level of genomic damage requires handling dozens of animals, which is difficult in the case of small populations. In this context, domestic dogs could provide a useful surrogate model because selection within dog breeds for desirable traits resulted in breeding individuals with similar morphological and physiological traits. Since their domestication, dogs have played important roles in human life, which include companionship, therapy support, hunting, protection of property and many other activities (Dreger et al. 2016a, b; Jansson and Laikre 2018).

Domestic dogs show a wide range of variations in the degree of inbreeding and lifespan (Yordy et al. 2020). To obtain animals with specific characteristics, such as body size, coat colour, and behavioural traits, a high number of dog breeds have been created by selective breeding (Jansson and Laikre 2018). Selective breeding leads to a rapid loss of genetic diversity in reducing heterozygosity (Leroy et al. 2011). Mellanby et al. (2013), evaluating the genetic diversity in 13 popular dog breed groups in the UK, found higher levels of homozygosity than in crossbred dogs, with the Golden retriever and Rottweiler showing the highest inbreeding levels, whilst the group with lowest level of inbreeding was represented by crossbred dogs. Similarly, the Norwegian Lundehund dog breed suffered a heavy loss of genetic diversity due to inbreeding (Kropatsch et al. 2015). These intensive selection practices have negatively impacted the genetic health of many purebred dogs, contributing to the inbreeding depression and increased occurrence of hereditary disorders associated with autosomal recessive alleles (Mellanby et al. 2013; Summers et al. 2010). Inbreeding depression impairs the vitality, performance and productivity functions of many animal populations (Chu et al. 2019). In dogs, inbreeding negatively influences reproduction and survival rates (Leroy et al. 2015). Another negative consequence of inbreeding is the high occurrence of physical diseases and genetic disorders amongst many dog breeds, which require frequent veterinary treatments (Olsson et al. 2011), with repercussions on animal welfare (Leroy et al. 2015).

The present research used a new approach based on a buccal micronucleus assay to evaluate the possible relationships between inbreeding and genomic damage using the dog as model species. In particular, we assessed the frequency of Micronuclei (MNi), nuclear buds (NBUDs) and other nuclear anomalies between purebred dogs and a control group represented by mixed breed animals.

Micronuclei represent small extranuclear bodies which have not been included in the daughter nuclei during telophase. They arise from chromosome breakage or a whole chromosome lag, which fails to be incorporated in one of the new nuclei (Krupina et al. 2021). Chromosomal instability was also measured by scoring NBUDs, i.e. nuclear protrusions, which represent the elimination process of amplified DNA and excess chromosomes from aneuploid cells (Bolognesi et al. 2013). Criteria for identifying and scoring cell types with MNi, NBUDs and other nuclear rearrangements are reviewed in Bolognesi et al. (2013) and Thomas and Fenech (2011).

The MNi assay is a very flexible, non-invasive, assay, widely used to evaluate genomic damage in humans and, recently, also in other non-human mammals, such as bats (Benvindo-Souz et al. 2019) and bottlenose dolphins (Gauthier et al. 1999), as well as in invertebrates (Santovito et al. 2020). The MNi assay also allows observing other nuclear anomalies, such as nuclei with condensed chromatin, nuclear indentation (nuclear invagination), pyknotic nuclei, and karyolitic cells that lose their nuclear material entirely. However, although these last nuclear anomalies represent structural anomalies associated with exposure to one or more environmental xenobiotics or with a specific physiological stress condition such as cell degeneration and apoptotic process (Thomas et al. 2009), they, differently to MNi and NBUDs, cannot be considered as index of genomic damage (Bolognesi et al. 2013). Moreover, this assay also allows recording the presence of binucleated cells, the excess of which is an indication of an imperfect cytodieresis mechanism.

## Materials and methods

### Sample collection

The study included 77 healthy purebred dogs and 75 healthy mixed breed dogs as a control group (Table 1).

**Table 1:** Mean (± SD) age and weight of sampled dogs according to breed and sex

	*N*	Age (years)	Weight (kg)
Pure-bred dogs			
Females	42	5.67 ± 2.67	16.60 ± 13.14
Males	35	6.23 ± 2.87	16.97 ± 11.51
Total	77	5.92 ± 2.76	16.77 ± 12.35
Mixed-bred dogs			
Females	37	5.68 ± 3.11	16.51 ± 9.98
Males	38	5.87 ± 2.78	18.18 ± 11.11
Total	75	5.77 ± 2.93	17.36 ± 10.53

All dogs lived in a human family context. Mixed breed dogs had been adopted from shelters, whereas purebred subjects were purchased from specialized breeders. No information was available about the origin of the mixed-bred dogs, thus, we cannot exclude their previous cross between pure breeds.

Amongst purebred dogs, we sampled the following breed (*n* = 7 for each breed) that represent the most frequently adopted purebred dogs: Golden Retriever, Jack Russel, German Shepherd, Dachshund, Poodle, Labrador, Chihuahua, Boxer, Border Collies, Bulldog and Pomeranian (Table 2).

**Table 2:** Results of statistical comparison between purebred and mixed bred dogs

Dogs	*N*	Cells	Micronuclei *N* (Mean ± S.D.)	Nuclear buds *N* (Mean ± S.D.)	Picnotic nuclei *N* (Mean ± S.D.)	Condensed chromatin *N* (Mean ± S.D.)	Indentation *N* (Mean ± S.D.)	Broken eggs *N* ((Mean ± S.D.)	Total aberrations *N* (Mean ± S.D.)	Binucleated cells *N* (Mean ± S.D.)
Pure-bred	77	77,000	173 (2.247 ± 1.359)^a^	156 (2.026 ± 1.597)^a^	144 (1.870 ± 2.975)^b^	74 (0.961 ± 2.314)^a^	51 (0.662 ± 1.401)^b^	29 (0.377 ± 0.844)^c^	627 (8.143 ± 5.460)^a^	84 (1.091 ± 1.687)
Golder retriever	7	7000	13 (1.857 ± 0.690)	20 (2.857 ± 1.676)	7 (1.000 ± 1.000)	1 (0.143 ± 0.378)	3 (0.429 ± 0.787)	0 (0.000 ± 0.000)	44 (6.286 ± 2.215)	10 (1.429 ± 2.149)
Jack Russel	7	7000	16 (2.286 ± 1.113)	13 (1.857 ± 1.952)	1 (0.143 ± 0.378)	22 (3.143 ± 3.671)	6 (0.857 ± 1.069)	7 (1.000 ± 1.528)	65 (9.286 ± 4.271)	14 (2.000 ± 3.215)
German shepherd	7	7000	19 (2.714 ± 2.059)	13 (1.857 ± 1.676)	26 (3.714 ± 6.448)	23 (3.286 ± 5.407)	3 (0.429 ± 0.787)	1 (0.143 ± 0.378)	85 (12.143 ± 10.668)	7 (1.000 ± 1.732)
Dachshund	7	7000	14 (2.000 ± 0.816)	13 (1.857 ± 0.900)	12 (1.714 ± 1.976)	9 (1.286 ± 2.215)	4 (0.571 ± 0.976)	1 (0.143 ± 0.378)	53 (7.571 ± 2.440)	6 (0.857 ± 1.574)
Poodle	7	7000	17 (2.429 ± 1.718)	11 (1.571 ± 0.976)	15 (2.143 ± 1.952)	2 (0.286 ± 0.488)	3 (0.429 ± 0.535)	4 (0.571 ± 0.787)	52 (7.429 ± 4.392)	13 (1.857 ± 2.340)
Labrador	7	7000	15 (2.143 ± 0.900)	10 (1.429 ± 1.397)	22 (3.143 ± 3.579)	2 (0.286 ± 0.756)	11 (1.571 ± 3.735)	6 (0.857 ± 1.215)	66 (9.429 ± 6.528)	5 (0.714 ± 0.488)
Chihuahua	7	7000	14 (2.000 ± 1.155)	17 (2.429 ± 2.573)	17 (2.429 ± 2.370)	6 (0.857 ± 0.900)	6 (0.857 ± 1.864)	4 (0.571 ± 1.134)	64 (9.143 ± 7.798)	6 (0.857 ± 1.215)
Boxer	7	7000	15 (2.143 ± 1.676)	12 (1.714 ± 1.113)	20 (2.857 ± 4.259)	4 (0.571 ± 0.976)	4 (0.571 ± 0.787)	0 (0.000 ± 0.000)	55 (7.857 ± 6.793)	5 (0.714 ± 0.951)
Border collies	7	7000	15 (2.143 ± 1.676)	18 (2.571 ± 2.370)	12 (1.714 ± 2.628)	3 (0.429 ± 1.134)	4 (0.571 ± 0.787)	2 (0.286 ± 0.488)	54 (7.714 ± 4.348)	3 (0.429 ± 0.787)
Bulldog	7	7000	16 (2.286 ± 1.113)	11 (1.571 ± 0.535)	3 (0.429 ± 0.535)	1 (0.143 ± 0.378)	5 (0.714 ± 0.951)	3 (0.429 ± 1.134)	39 (5.571 ± 1.512)	12 (1.714 ± 1.604)
Pomeranian	7	7000	19 (2.714 ± 1.976)	18 (2.571 ± 1.618)	9 (1.286 ± 1.799)	1 (0.143 ± 0.378)	2 (0.286 ± 0.488)	1 (0.143 ± 0.378)	50 (7.143 ± 2.673)	3 (0.429 ± 0.787)
Mixed-bred	75	75,000	53 (0.707 ± 0.802)	74 (0.987 ± 1.214)	49 (0.653 ± 1.121)	23 (0.307 ± 0.592)	13 (0.173 ± 0.381)	6 (0.080 ± 0.273)	218 (2.907 ± 1.890)	100 (1.333 ± 2.069)

*N* number of analysed subjects, *SD* standard deviation

^a^*P* < 0.001

^b^*P* = 0 < 0.01

^c^*P* < 0.05, with respect to mixed-bred group (Mann–Whitney test)

Data about age, sex and weight, obtained by dog owners for purebred and by veterinarians for mixed breed, were collected to evaluate their possible influence on the level of genomic damage. It is well known that drugs and X-rays can alter the level of genomic damage (Santovito et al. 2014a, b). Therefore, we excluded from the study subjects that had contracted acute infections and chronic non-infectious diseases or were exposed to diagnostic X-rays for a minimum of 1 year before the analysis. Similarly, we excluded from the sampling subjects who showed infections or pathologies affecting the oral cavity diagnosed by the competent veterinarians.

### Buccal MNi assay

Buccal MNi assay was performed as described in Santovito et al. (2022a, b) with few modifications. Briefly, exfoliated buccal mucosa cells were collected by gently scraping the mucosa of the inner lining of one or both cheeks with a toothbrush (Figure S1—Supplementary Materials 1). Buccal cells were also collected from the inner side of the lower lip and palate. Indeed, the variation in MNi frequency between these areas was minimal for control subjects, as demonstrated in Holland et al. 2008. The toothbrush tip was immersed in a fixative solution consisting of methanol/acetic acid 3:1, shaken for at least 1 min and stored at 4 °C before the analysis. Successively, cells were collected by centrifugation, the supernatant was discarded, and the pellet was dissolved in a minimal amount of fixative, which was seeded on the slides to detect MNi by conventional staining with 5% Giemsa (pH 6.8) prepared in Sörensen buffer. Microscopic analysis was performed at 1000X magnification on a light microscope. According to the established criteria, MNi, NBUDs and other nuclear rearrangements were scored in 1000 cells with well-preserved cytoplasm per subject (Thomas et al. 2009). In the count of the total number of nuclear aberrations, we excluded the binucleated cells, as they do not represent genomic damage.

The slides were read by three expert evaluators who were unaware of the analyzed individual and the group to which he belonged, as the initials placed on the slide had previously been hidden. All possible cases of genomic damage (MNi and NBUDs) observed by microscope were photographed and collectively evaluated by computer.

### Statistical analysis

Counts of micronuclei and other abnormalities are presented as the mean frequency (± standard deviation) in a sample of 1000 cells/subject. Since data were skewed and not normally distributed (Supplementary materials 2), we used the Mann–Whitney test to compare two sample groups and the Kruskal–Wallis test for more than two groups. No significant statistical differences were found amongst different breeds in MNi and NBUDs frequencies (Table 2); therefore, they were pooled in a single purebred sample for further analyses.

The statistical differences between the number of males and females belonging to the studied groups were evaluated by the Fisher's Exact Chi-square test.

Since the response variables were count data, we modelled the effect of predictive variables on MNi and NBUDs using Generalized Linear Models (GLMs) with a Poisson distribution. We limited the GLMs analysis to MNi and NBUDs because they represent indexes of genomic damage (anaeugenic/clastogenic damage and gene amplification, respectively), whereas the other nuclear aberrations are associated to cell degeneration and apoptotic processes (Thomas et al. 2009).

We used GLMs to evaluate if abnormalities frequencies were influenced by breed type, sex, index (as factors), age and weight (covariates) considering main effects and the possible interaction between sex and age. We used the ratio variance/mean and the overdispersion parameter as the scaled Pearson’s *χ*^2^ estimated and explored the residuals of the full model for influential points and outliers to evaluate data dispersion. In case of over or underdispersion, data were modelled with a negative binomial distribution with a log link. We modelled the main effects and the interaction between age—sex, and breed—stress. All the analyses were performed using R software (R Core Team, version 4.2.2., 2022).

## Results

We sampled 77 purebred dogs (mean age: 5.92 ± 2.76, mean weight: 16.77 ± 12.35, 42 females and 35 males) and 75 mixed-bred dogs (mean age: 5.77 ± 2.93, mean weight 17.36 ± 10.53, 37 females and 38 males) (Table 1).

No significant differences were found between the two groups in terms of mean age (purebred 5.92 ± 2.75, mixed bred 5.77 ± 2.93, *U* = 3063, *p* = 0.52), mean weight (purebred 16.77 ± 12.35, mixed bred 17.36 ± 10.53, *U* = 2758, *p* = 0.63) and the number of males and females (purebred 42 females and 35 males, mixed bred 37 females and 38 males, Fisher’s Exact Test, *p* = 0.63).

**Fig. 1:**
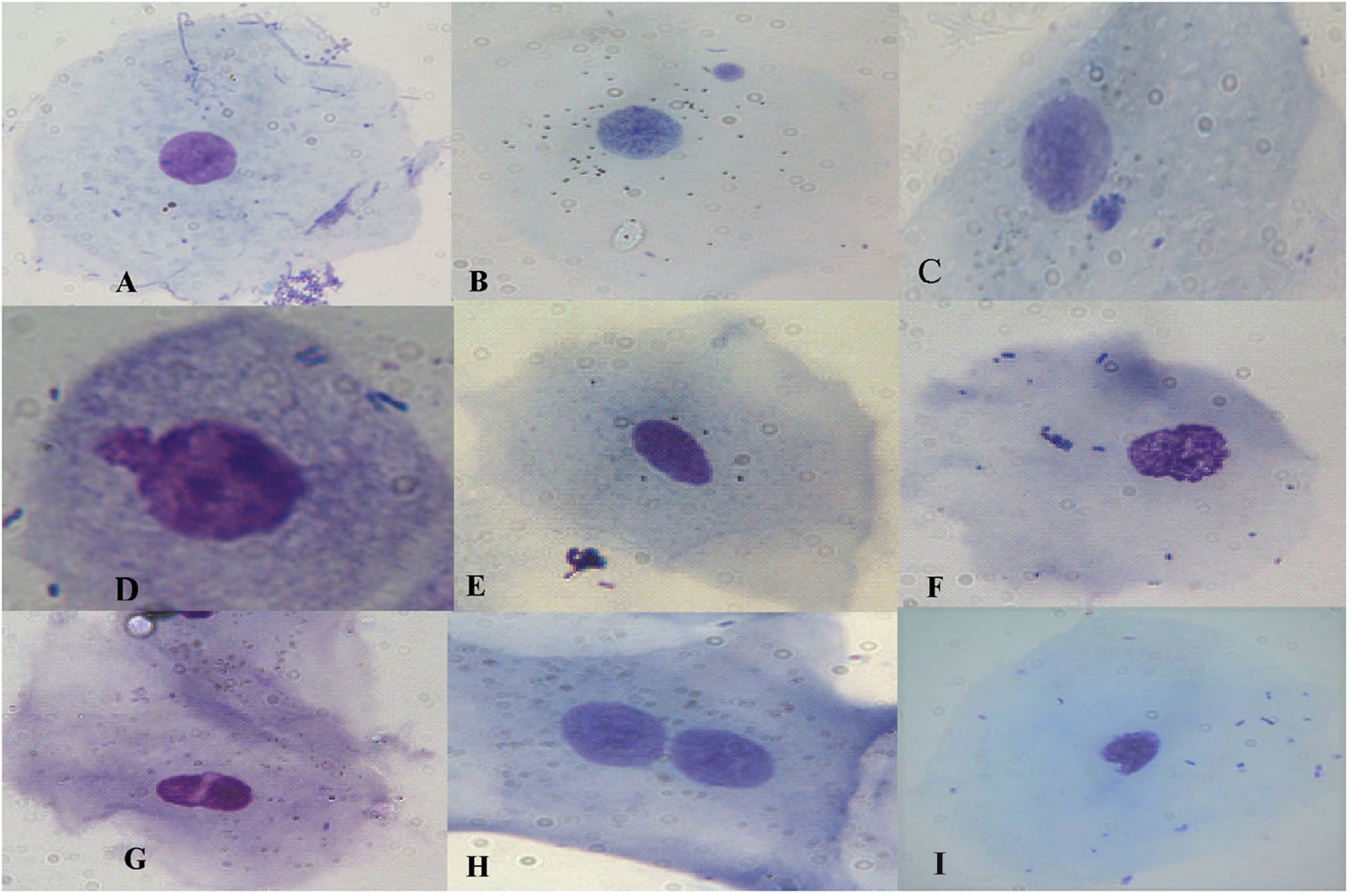
Examples of genomic damage observed in pure-bred and mixed-bred dogs. **A** normal cell, **B**, **C** Cell with micronucleus, **D** nuclear bud, **E** picnotic nucleus, **F** condensed chromatin, **G** broken eggs, **H** binucleated cell, **I** indentation (invagination)

We read 152,000 cells (77,000 for purebred dogs and 75,000 for mixed-bred dogs). Some examples of damaged cells observed in our samples are reported in Fig. 1.

Significant differences were found between purebred and mixed-bred dogs in terms of MNi, NBUDs, Picnotic Nuclei, Condensed Chromatin, Indentation, Broken Eggs and Total Nuclear Aberrations, with the purebred dogs showing the highest values (Table 2). Vice versa, no significant differences in the frequency of micronuclei and other nuclear aberrations were found between the different breeds (Table 2).

We used a Poisson GLM for MNi and NBUDs. The breeding condition was the only variable influencing MNi ratio (Estimate = 1.131 ± 0.1996, Table 3). The incident rate ratio [Exp(B) = 3.100 (95%CI 2.132—4.657)] indicated that the MNi frequency was 210% higher in purebreds than mixed-bred dogs. NBUDs data were overdispersed; therefore, the GLM was modelled with a negative binomial distribution. Also for NBUDs ratio the breeding condition was the only selected variable (Estimate = 0.818 ± 0.149; Table 2) and was influenced by breeding condition (*B* = 0.800 ± 0.198) and the stress index (*B* = 0.342 ± 0.164). The incidence rate ratio [Exp(B) = 2.266 (95%CI 1.652–3.139] indicate a frequency of this damage that is 123% higher in purebred with respect to mixed-bred dogs (Table 3). The power of the models was 0.959 for MNi and 0.954 for NBUDs.

**Table 3:** Full Poisson (MNi) or negative binomial (NBUDs) GLM results evaluating effects of covariates on MNi and NBUDs frequencies in pure and mixed breed dogs

	Estimate	SE	z value	Pr( >|z|)	Exp(B)	Wald CI 95%
MNi: MNi ~ breed + weight + age + sex + age * sex						
Intercept	−0.570	0.276	−2.063	0.039	0.566	0.324–0.958
Breed [purebred]	1.131	0.199	5.695	0.000	3.100	2.132–4.657
Weight	0.003	0.006	0.451	0.652	1.003	0.991–1.014
Age	0.036	0.033	1.115	0.265	1.037	0.972–1.104
Sex [males]	0.018	0.327	0.055	0.956	1.018	0.533–1.923
Age:sex [males]	−0.021	0.048	−0.437	0.662	0.979	0.891–1.077
NBUDs: NBUDs ~ breed + weight + age + sex + age * sex						
Intercept	−0.218	0.256	−0.851	0.395	0.804	0.483–1.314
Breed [purebred]	0.818	0.164	5.002	0.000	2.266	1.652–3.139
Weight	0.005	0.006	0.737	0.461	1.005	0.992–1.017
Age	0.027	0.032	0.845	0.398	1.028	0.964–1.094
Sex [males]	−0.304	0.353	−0.862	0.388	0.738	0.366–1.460
Age:sex [males]	−0.027	0.052	−0.512	0.609	0.974	0.879–1.079

## Discussion

Due to their population structure, domestic dogs represent a valuable model for studying the effect of inbreeding in small populations. Indeed, despite the sizeable total dog population, reproductive population sizes for purebred dogs are often small. Moreover, reproduction within breeds is usually tightly controlled, and populations of free-breeding dogs can be used as control populations in genetic studies (Yordy et al. 2020).

One of the most critical concerns in purebred dogs is the high occurrence of genetic disorders and physical diseases that may affect animals' survival rates (Leroy et al. 2015). A potential cause of these problems is inbreeding, which is known to reduce the viability of individuals and the within-population genetic diversity, with the consequent increase in the incidence of inherited diseases (Mellersh 2008; Lewis et al. 2015). In this human selective pressure context, the crossing between close relatives was often carried out without considering the potentially deleterious effects associated with the simultaneous loss of genetic diversity and the potential increase of “deleterious” allele frequencies (Dreger et al. 2016a, b).

In the present paper, we used a new, non-invasive, approach based on the buccal micronucleus assay to evaluate the possible variation in genomic instability across different dog breeds, using the dog as model species. We found a significant increase of MNi and NBUDs frequencies in purebred dogs with respect to mixed bred dogs, suggesting a possible relationship between inbreeding and the level of genomic damage. No data are present in literature about MNi frequency in wild dogs, thus, it is impossible for us to compare our results. However, the frequency of MNi observed in our purebred dogs is significantly higher with respect to that observed in a previously study for wild boar (0.512 ± 0.597) (Santovito et al. 2022a, b). In humans, the highest MNi frequency levels were associated with increased incidence of cancer, cardiovascular and neurological diseases, and, consequently, a reduction of life expectancy (Fenech et al. 2021). This correlation is so strong that the micronucleus assay can be used as a predictor against some tumour pathologies, as observed in the case of bladder, pulmonary and cervical cancer (Pardini et al. 2017; Asanov et al. al. 2021; Setayesh et al. 2020). Similarly, also for dogs, we can hypothesize an association between the highest levels of MNi and a reduction in longevity due to a higher incidence of genetic disorders. This could explain, for example, that purebred dogs, showing high levels of inbreeding, usually have a higher incidence rate of those disorders, such as cancer, which may significantly reduce their lifespan than mixed breed dogs (Proschowsky et al. 2003; Klopfenstein et al. 2016). This reduced longevity in purebred dogs was linked to increased early mortality, early onset of senescence and increased rate of ageing (Kraus et al. 2013; Leroy et al. 2015). In a study conducted on the Bernese Mountain Dog, the main pathologies underlying a low life expectancy were neoplasms and degenerative diseases (Proschowsky et al. 2003). Yordy et al. (2020) observed that some purebred dogs had an average age at death between seven (Bernese, Hounds and Molossers) and 10 years (Poodles and Shepherds), whilst in mixed breed dogs, this increased up to 11–12 years.

It is difficult to explain the mechanisms behind this significant increase in MNi in purebred dogs. However, this highest incidence could be related to the reduced efficiency of DNA-repair mechanisms. This may depend on an increase in the frequencies of minor alleles of those polymorphic genes belonging to Base Excision Repair and Nucleotide Excision Repair systems as a consequence of an increase in homozygote genotypes and a decrease in genetic diversity (Mellanby et al. 2013). Indeed, the possible association between these minor alleles and increased levels of genomic damage was demonstrated by different authors (Santovito et al. 2017; Zhang et al. 2019).

Although it is known that there is a great variation in inbreeding both across and within breeds (Yordy et al. 2020; Jansson and Laikre 2018; Leroy et al 2015), and that some breeds are apparently not inbred (Mellanby et al 2013), no differences we found amongst the different breeds in terms of MNi and NBUDs (Table 2). The absence of significant differences in the frequency of micronuclei amongst different breeds reinforces the hypothesis that the observed increased genomic damage amongst purebred dogs may not be due to the different genomic instability typical of a particular breed, but to inbreeding itself.

However, we would like emphasize that purebred dogs can differ from mixed breed dogs in terms of early puppyhood environment, likelihood of being spayed or neutered, and reproductive history, each of which could represent a confounding factor that could affect the level of genomic damage. Nevertheless, purebred dogs are generally recruited from specialized farms where the puppy, siblings and mother are kept together. Thanks to the European legislation on animal welfare (EU Platform on Animal Welfare 2020), the separation of the puppy from the mother cannot occur before two months of age, allowing the dog to develop correctly. In addition, purebred dogs are generally raised at home as active members of the family group, which means that the environment in which the animal lives is very different from the conditions of mixed-breed dogs. The latter often come from kennels, shelters or stray conditions, in which sensory deprivation, mistreatment and the non-satisfaction of ethological and physiological needs can lead to higher levels of stress and genomic damage (Santovito et al. 2022a, b; Dalla Villa et al. 2013). The latter data, therefore, seem to reinforce our finding.

Moreover, it is known that there is substantial variation in inbreeding across dog breeds and across individuals within a breed. Indeed, comparing the F_adj_ inbreeding values obtained by Bannasch et al. (2021) to each of the breeds analyzed in the present study (Supplementary Material 3), we can observe high variable values, ranging from 0.104 for Jack Russell Terrier to 0.395 for Boxer, a much larger spread than the difference in mean inbreeding between purebred dogs overall and mixed breed dogs. Highly variable values were also described by Dreger et al. (2016a, b) that, comparing the WGS and SNP data across 50 breeds, observed a range of F-value calculated from the WGS of 0.488, from a minimum of 0.084 for Beagle to a maximum of 0.571 for Basenji, and a range in SNP-based F-values of 0.423, from 0.113 for Chihuahua to 0.536 for Basenji.

Despite this highly variable Fadj range, in our work we did not observe a significant difference, in the level of genomic damage, between different breeds (Supplementary Material 3). A possible explanation of this result is that *F*_adj_ values from Bannasch et al. (2021) might not be representative of purebred dog populations in northern Italy. In this sense, the individual consanguinity values obtained by genomic analysis (GWA and SNP analysis) could be better highlight the possible correlation between genomic damage and the inbreeding levels (Dreger et al. 2016a, b). This represents a limitation of the present paper. However, our aim, rather than establishing a causal correlation between inbreeding and genomic damage, was to compare the levels of genomic damage between purebred and mixed dogs, net of any other confounding factor. As far as we are concerned, inbreeding depression could be a possible explanation of the observed results, although our study does not demonstrate any correlation or direct causality”.

Sex and age were not found to influence the frequency of genomic damage (Table 3 and Figure S2—Supplementary Materials 4). The absence of a sex effect is in accordance with previous studies on shelter dogs and cats (Santovito et al. 2022a, b), on bats (Benvindo-Souz et al. 2019) and on the bottlenose dolphin *Tursiops truncatus* (Zamora-Perez 2006). However, different frequencies of MNi and NBUDs between males and females have been reported in humans (Santovito and Gendusa 2020; Gajski et al. 2018).

Unlike what was found in dogs, an effect of age on the frequency of MNi was found in humans (Thomas et al. 2008; Santovito and Gendusa 2020). This is probably due to dogs’ relatively short life expectancy, which may mask any possible correlation between age and damage frequency. Our results are in line with those obtained by Zamora-Perez et al. (2006) and by Zúñiga-González et al. (2001), which evaluated a possible relationship between genomic damage and age in dolphins and squirrels, although on erythrocytes and not on buccal cells.

Finally, the absence of a weight effect on genomic damage frequencies contrasts with Middleton et al. (2017) and Jimenez and Downs (2020) results. These authors detected lower total concentrations of antioxidants such as glutathione, urate, bilirubin and catalase, with a consequent higher concentration of free radicals and higher levels of genomic and lipidic damage in smaller dogs than large-size dogs, as a consequence of the greatest basal metabolic activity of the former. Interestingly, a general reduction of antioxidant capacities has been observed in domestic animals and has been attributed to the adverse effects of artificial selection (Jimenez and Downs 2020). This finding partially explains the shorter lifespan of domestic dogs compared to the potential life spans of wolves (Jimene and Downs 2020).

## Conclusions

Natural populations are increasingly fragmented by habitat loss. One of the paradigms of conservation biology is that small populations experience reduced viability due to loss of genetic diversity and inbreeding, that can increase their risk of extinction. Indeed, isolation and small population size are thought to reduce individual and population fitness via inbreeding depression. The latter can reduce individual fitness and contribute to extinction of wild and captive populations.

Results of the present paper represent the first demonstration, to our knowledge, that inbreeding could also affect the levels of genomic damage, in terms of increased frequencies of MNi and NBUDs. It is our opinion that, considering the association between genomic damage, the reduction of the survival expectancy and the increase of health problems affecting animal welfare, the results we obtained may represent a stimulus to intensifying genetic restoring policies by immigration of unrelated individuals in wild populations, and to discourage the use of intensive inbreeding practices and mating between close relatives in captive populations. In this scenario, the development of new assays, including MNi assay, could improve our capacity to evaluate the consequences of inbreeding and, in combination with more traditional genetic and analytical techniques, could provide a more complete picture of the health status of many animal species.

### Supplementary Information

Below is the link to the electronic supplementary material.

Supplementary file1 (DOCX 159 KB)

Supplementary file2 (DOCX 16 KB)

Supplementary file3 (DOCX 14 KB)

Supplementary file4 (DOCX 1308 KB)

## Data Availability

The datasets generated during and/or analysed during the current study are available from the corresponding author on request.
