# Supplementary material for: Purebred dogs show higher levels of genomic damage compared to mixed breed dogs
Source: Mamm Genome. 2023 Oct 21;35(1):90–8. doi: 10.1007/s00335-023-10020-5 (PMC10884103; doi:10.1007/s00335-023-10020-5)
Supplement: Supplementary file 1 — Supplementary file1 (DOCX 159 KB) [file 335_2023_10020_MOESM1_ESM.docx]

**Supplementary Material 1**


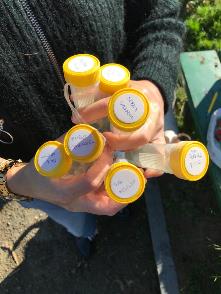

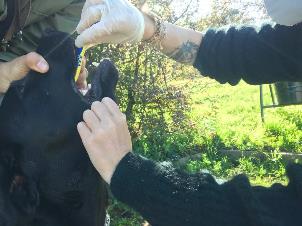

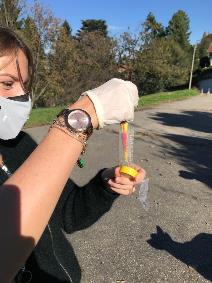


**A**

**B**

**C**

Figure S1. Representation of the first 3 steps of buccal cell collection. A = Preparation of marked Falcon with 15-20 mL of fixative solution (Methanol-Acetic Acid 3:1); B = Collection of buccal cells from the inner side of the lower lip and palate; C = Immersion and shaking of the tip of the spatula, for at least 1 min, in the fixative solution.
