# Supplementary material for: Purebred dogs show higher levels of genomic damage compared to mixed breed dogs
Source: Mamm Genome. 2023 Oct 21;35(1):90–8. doi: 10.1007/s00335-023-10020-5 (PMC10884103; doi:10.1007/s00335-023-10020-5)
Supplement: Supplementary file 2 — Supplementary file2 (DOCX 16 KB) [file 335_2023_10020_MOESM2_ESM.docx]

**Supplementary Material 2 – Data distribution**

|  | Group | Kolmogorov-Smirnov Shapiro-Wilk | | | |
| --- | --- | --- | --- | --- | --- |
|  |  | Statistics | Sign. | Statistics | Sign. |
| Age | 1 | .120 | **.008** | .960 | **.017** |
|  | 2 | .121 | **.008** | .932 | **<.001** |
| Weight | 1 | .216 | **<.001** | .886 | **<.001** |
|  | 2 | .224 | **<.001** | .882 | **<.001** |
| MNI | 1 | .247 | **<.001** | .893 | **<.001** |
|  | 2 | .264 | **<.001** | .759 | **<.001** |
| NBUDs | 1 | .221 | **<.001** | .904 | **<.001** |
|  | 2 | .269 | **<.001** | .762 | **<.001** |
| Picnotic  Nuclei | 1 | .303 | **<.001** | .665 | **<.001** |
|  | 2 | .387 | **<.001** | .640 | **<.001** |
| Condensed  Chromatin | 1 | .375 | **<.001** | .481 | **<.001** |
|  | 2 | .458 | **<.001** | .561 | **<.001** |
| Indentation | 1 | .318 | **<.001** | .495 | **<.001** |
|  | 2 | .502 | **<.001** | .458 | **<.001** |
| Broken  Eggs | 1 | .452 | **<.001** | .515 | **<.001** |
|  | 2 | .535 | **<.001** | .301 | **<.001** |
| Binucleated  Cells | 1 | .274 | **<.001** | .689 | **<.001** |
|  | 2 | .327 | **<.001** | .696 | **<.001** |
| Total  Aberrations | 1 | .201 | **<.001** | .883 | **<.001** |
|  | 2 | .173 | **<.001** | .861 | **<.001** |

1= Pure-Bred dogs group

2 = Mixed-Bred dogs group
