# Supplementary material for: Purebred dogs show higher levels of genomic damage compared to mixed breed dogs
Source: Mamm Genome. 2023 Oct 21;35(1):90–8. doi: 10.1007/s00335-023-10020-5 (PMC10884103; doi:10.1007/s00335-023-10020-5)
Supplement: Supplementary file 3 — Supplementary file3 (DOCX 14 KB) [file 335_2023_10020_MOESM3_ESM.docx]

Supplementary Material 3 – Number of MNi and NBUDs associated to F_adj_ values obtained from Bannash et al (2021).

| **Breed** | **F_adj *_** | **MNi** | **NBUDs** | **Total genomic damage** |
| --- | --- | --- | --- | --- |
| Border Collies | 0.195 | 15 | 18 | 33 |
| Boxer | 0.357 | 15 | 12 | 27 |
| Bulldog | 0.348 | 16 | 11 | 27 |
| Chihuahua | 0.111 | 14 | 17 | 31 |
| Dachshund | 0.247 | 14 | 13 | 27 |
| German Shepherd | 0.287 | 19 | 13 | 32 |
| Golder Retriver | 0.237 | 13 | 20 | 33 |
| Jack Russel | 0.104 | 16 | 13 | 29 |
| Labrador | 0.217 | 15 | 10 | 25 |
| Pomeranian | 0.136 | 19 | 18 | 37 |
| Poodle | 0.149 | 17 | 11 | 28 |

*data obtained from Bannash et al. (2021); **F_adj_** = adjusted inbreeding coefficient;

MNi = micronuclei; NBUDs = nuclear buds
