# Supplementary material for: Purebred dogs show higher levels of genomic damage compared to mixed breed dogs
Source: Mamm Genome. 2023 Oct 21;35(1):90–8. doi: 10.1007/s00335-023-10020-5 (PMC10884103; doi:10.1007/s00335-023-10020-5)
Supplement: Supplementary file 4 — Supplementary file4 (DOCX 1308 KB) [file 335_2023_10020_MOESM4_ESM.docx]

**Supplementary Material 4 - Frequency of abnormalities according to sex.**


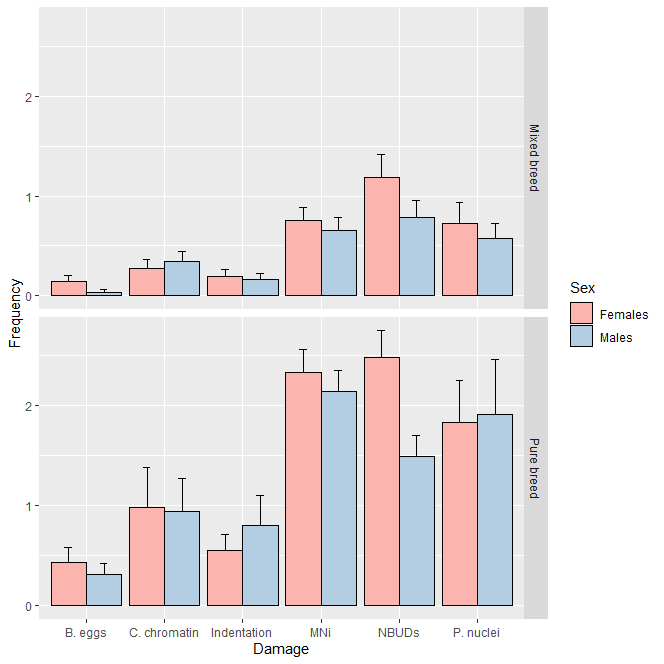


**Figure S2**. Frequency of abnormalities (mean ± SE in 1,000 cells/individual) in pure and mixed breed dogs according to sex. B. eggs = Broken eggs; C. chromatin = Condensed chromatin, MNi = Micronuclei, NBUDs = Nuclear Buds, P. nuclei = Picnotic nuclei
